# Parallels in Sepsis and COVID-19 Conditions: Implications for Managing Severe COVID-19

**DOI:** 10.3389/fimmu.2021.602848

**Published:** 2021-02-03

**Authors:** Charles Ochieng’ Olwal, Nora Nghuchuzie Nganyewo, Kesego Tapela, Alexandra Lindsey Djomkam Zune, Oloche Owoicho, Yaw Bediako, Samuel Duodu

**Affiliations:** ^1^West African Centre for Cell Biology of Infectious Pathogens (WACCBIP), University of Ghana, Accra, Ghana; ^2^Department of Biochemistry, Cell and Molecular Biology, College of Basic and Applied Sciences, University of Ghana, Accra, Ghana

**Keywords:** coronavirus, COVID-19, SARS-CoV-2, sepsis, cytokine storm, immunosuppression, hypovolemia

## Abstract

Sepsis is a life-threatening systemic illness attributed to a dysregulated host response to infection. Sepsis is a global burden killing ~11 million persons annually. In December 2019, a novel pneumonia condition termed coronavirus disease 2019 (COVID-19), caused by severe acute respiratory syndrome coronavirus 2 (SARS-CoV-2), emerged and has resulted in more than 1,535,982 deaths globally as of 8^th^ December 2020. These two conditions share many pathophysiological and clinical features. Notably, both sepsis and COVID-19 patients experience consumptive thrombocytopenia, haemolytic anaemia, vascular microthrombosis, multi-organ dysfunction syndrome, coagulopathy, septic shock, respiratory failure, fever, leukopenia, hypotension, leukocytosis, high cytokine production and high predisposition to opportunistic infections. Considering the parallels in the immunopathogenesis and pathophysiological manifestations of sepsis and COVID-19, it is highly likely that sepsis care, which has a well-established history in most health systems, could inform on COVID-19 management. In view of this, the present perspective compares the immunopathogenesis and pathophysiology of COVID-19 and non-SARS-CoV-2 induced sepsis, and lessons from sepsis that can be applicable to COVID-19 management.

## Introduction

Sepsis is a life-threatening systemic illness associated with a dysregulated host response due to invasion of the bloodstream by pathogen(s), such as bacteria, viruses, fungi or parasites (1). Global estimates show that about 49 million people are affected by sepsis, contributing potentially to 11 million deaths annually (2). Sepsis remains one of the leading causes of preventable deaths in all age groups (3) and gender. Thus, sepsis is considered a health priority by the World Health Organization (WHO), which has provided recommendations on how to improve prevention, diagnosis and management of sepsis (4).

In December 2019, a novel pneumonia condition termed coronavirus disease 2019 (COVID-19), caused by severe acute respiratory syndrome coronavirus 2 (SARS‐CoV‐2), with many pathophysiological and clinical parallels to sepsis was reported in Wuhan, China (5). COVID-19 spread rapidly and was declared a global pandemic by WHO on 11^th^ March 2020. One year later, over 66.72 million confirmed cases and 1.53 million fatalities related to COVID-19 have been reported (https://www.who.int/emergencies/diseases/novel-coronavirus-2019).

Clinical manifestations of sepsis begin with inflammation and progress to circulatory organ dysfunction associated with significant haematopathologic changes. Well-defined clinical features include consumptive thrombocytopenia, haemolytic anaemia, vascular microthrombosis (1), multiorgan dysfunction syndrome (6), coagulopathy (7) and septic shock. Other manifestations include increased heart rate, respiratory failure, fever, leukopenia, hypotension (8), leukocytosis, cytokine storm, and high predisposition to opportunistic infections (9). Strikingly, these manifestations are also common to COVID-19 (10, 11).

Considering the parallels between COVID-19 and non-SARS-CoV-2 induced sepsis (hereafter referred to as sepsis), it is possible that the two conditions could impact one another. Here, we compare the immunopathogenesis and pathophysiology of COVID-19 and sepsis, and the potential lessons from sepsis management that may be applicable to COVID-19 management.

## Immunopathogenesis and Pathophysiology of COVID-19 and Sepsis

### COVID-19

Initial studies demonstrated that COVID-19 severity is directly correlated with serum levels of pro-inflammatory cytokines, including interleukin (IL) 6 (IL-6) and IL-1 beta (IL-1β), as well as IL-2, IL-8, IL-17, granulocyte colony-stimulating factor (G-CSF), granulocyte macrophage-colony stimulating factor (GM-CSF), human interferon-inducible protein 10 (IP10), monocyte chemotactic protein-1 (MCP1), chemokine (C-C motif) ligand 3 (CCL3), and tumour necrosis factor (TNF) (12, 13). It was suggested that these elevated levels of pro-inflammatory cytokines termed cytokine storm, may result in shock and tissue damage, leading to multiple organ failure (14). The release of cytokines also contribute to massive pulmonary pathology, resulting in enormous infiltration of neutrophils and macrophages, diffuse alveolar damage with the development of hyaline membranes and thickening of the alveolar wall (15). Spleen atrophy and lymph node necrosis have also been observed during autopsy in deceased COVID-19 patients, suggestive of immune-mediated damage (14). However, recent studies are beginning to challenge the centrality of cytokine storm in severe COVID-19 cases. For instance, a study demonstrated that inflammatory cytokine elevations in patients with severe and critical COVID-19, including elevations of IL-6, are profoundly lower than those reported in patients with comparable conditions such as acute respiratory distress syndrome (ARDS), which is unrelated to COVID-19 and sepsis. In the same study, several non-cytokine biomarkers, including D-dimer, C-reactive protein, and ferritin, were elevated to a similar or greater extent in COVID-19 patients than in patients with sepsis or ARDS (16). No statistically significant differences were observed in baseline levels of multiple cytokines between COVID-19 and sepsis patients (17). The COVID-19 complications are now largely being associated with immunosuppression (18), especially lymphopenia rather than cytokine storm (12, 19). Increased T lymphopenia, particularly CD4+ and CD8+ T cells have been shown to decrease markedly in COVID-19 patients, implying damage of lymphocytes by the SARS-CoV-2 virus (13, 20, 21). Moreover, unlike cytokine storm which is episodic, lymphopenia is continuous in critically ill COVID-19 patients and leads to increased secondary infections (22). Considering the limitations of the previous studies such as modest sample sizes and non-inclusion of cohorts exhibiting cytokine storm-mediated hyperinflammation with accompanying lung and organ injury (18), more investigations are required to firmly confirm whether it is the cytokine storm or immunosuppression that drives unrestrained viral dissemination and organ injury observed in severe COVID-19 patients.

### Sepsis

Upon bacterial infection, pattern recognition receptors (PRRs), such as toll-like receptors, bind to the pathogen-associated molecular patterns and danger-associated molecular patterns expressed on pathogens to initiate intracellular signaling within cells (23). PRRs expressed on the surface of monocytes and neutrophils activate the intracellular kinase pathways, which induce activation of nuclear factor-kappaB (NF-κB) in the cytoplasm (24). NF-κB is then translocated to the nucleus to upregulate the pro-inflammatory response to eliminate the invading pathogen(s) (24). The pro-inflammatory for consistency responses include leukocyte activation, cytokine production, reactive oxygen species, inducible nitric oxide synthase (iNOS) activation, complement, coagulation activation, and enzymatic activation of cellular proteases (25). However, despite this repertoire of responses, pathogens can persist in the blood, resulting in complex interactions with the immune system leading to sepsis. Sepsis is characterized by an imbalance between pro- and anti-inflammatory responses (26). The intense production of pro-inflammatory mediators can result in cytokine storm, which deregulates the immune response and activates pathological inflammatory disorders (27).

The dysregulated immune response and production of nitric oxide (NO) associated with sepsis, may progress to vascular endothelial injury and circulatory organ dysfunction associated with significant haematopathologic changes (1). Overproduction of NO also leads to persistent vasodilation, which affects normal blood flow to organs; contributing to hypotension and hypoxia. Hypotension and hypoxia harm organs such as the brain, pancreas, liver, and mitochondria causing multiple organ dysfunction syndrome (1). Studies have shown that vascular endothelial injury leads to acute lung injury and respiratory distress syndrome (28). C3a and C5a from the complement system have been associated with strong upregulation of coagulation factors, resulting in disseminated intravascular coagulation (DIC). Prolonged activation of NF-κB and reduced caspase 3 levels delay the apoptosis of activated macrophages and neutrophils, resulting in organ injury and immunesuppression (29). Immunesuppression during sepsis involves reduced expression of Human Leukocyte Antigen (HLA)–DR isotype on blood monocytes and upregulation of anti-inflammatory cytokines (IL-10), which causes immunoparalysis (30). Immunosuppression promotes opportunistic pathogen invasion such as seen with nosocomial infections (1).

## Clinical Manifestations and Pathophysiology of COVID-19 and Sepsis: Overlaps and Deviations

Clinical manifestations of COVID-19 are classified as mild, moderate, severe, or critical (31). Mild infection involves symptoms such as dry cough, nasal congestion, sore throat, loss of taste and/or smell, mild fever, headache, malaise, muscle pain, and vomiting (32). The moderate form of the disease involves shortness of breath or tachypnea in children, while severe cases include fever associated with respiratory distress, hypoxia, dyspnea, and tachypnea (32). However, in critical cases, patients suffer from respiratory failure, septic shock, as well as multiple organ dysfunction or failure (33).

Like sepsis, COVID-19 symptoms include elevated bilirubin, hypoxia, reduced glomerular filtration rate, and hypoalbuminemia (34, 35). Coronary heart disease, chronic renal failure and dementia are also common in both COVID-19 and sepsis patients (36). Moreover, DIC and abnormal coagulation associated with sepsis have also been observed in severe COVID-19 (37). Although the mechanisms of coagulopathy in critically ill COVID-19 patients are yet to be determined, it has been suggested that SARS-CoV-2 attacks vascular endothelial cells [reviewed in (38)], leading to abnormal coagulation. The haematological phenotype of COVID-19-induced coagulopathy is somewhat different from that in typical sepsis. Whereas sepsis is characterized by systemic hypercoagulation and suppressed fibrinolysis, severe COVID-19-induced coagulopathy promotes local thrombus formation (39). In addition, venous thromboembolism and arterial thrombosis occur more frequently in COVID-19-induced coagulopathy compared to non-SARS-CoV-2 induced coagulopathy [reviewed in (40)]. Further, COVID-19 complications include acute respiratory failure and cytokine storm, which are capable of damaging organs (37). Notably, according to the Sepsis-3 International Consensus, patients with the above complications meet the diagnostic criteria for sepsis and septic shock (41), implying that the SARS-CoV-2 infection predisposes people to sepsis. Indeed, the Global Sepsis Alliance has stated that SARS-CoV-2 causes sepsis (42). This is supported by a recent study in which viral sepsis was reported as the most frequent complication in COVID-19 and is associated with high mortality of severe COVID-19 patients (37). **Table 1** summarizes the similarities and differences between sepsis and COVID-19 conditions.

**Table 1:** Similarities and differences between COVID-19 and Sepsis conditions.

Similarities	References	Differences	References
Multiple organ dysfunction	(1, 14)	Venous thromboembolism and arterial thrombosis occur more frequently in COVID-19-induced coagulopathy compared to non-SARS-CoV-2 induced coagulopathy	(40)
Immunosuppression	(18, 29)	Cytokine storm is the major problem in sepsis while, immunosuppression is the major problem in COVID-19.	(18)
Disseminated intravascular coagulation (DIC) and abnormal coagulation	(37, 39)	Sepsis is characterized by systemic hypercoagulation and suppressed fibrinolysis while severe COVID-19 induced coagulopathy promotes local thrombus formation	(39)
Elevated bilirubin, hypoxia, reduced glomerular filtration rate, and hypoalbuminemia	(34, 35)	SARS-CoV-2 infection predisposes people to sepsis, but sepsis may not be a risk factor to SARS-CoV-2	(42)
Acute respiratory failure and cytokine storm	(27, 37)		

## Approaches for Managing Severe COVID-19: Lessons From Sepsis

Barely three months into the COVID-19 pandemic, a number of guidelines were issued for or against the management of critically ill adult COVID-19 patients (43). Previously, WHO had issued guidelines on how to manage sepsis menace (4), although it still remains a health challenge. Here, leveraging the striking similarities between severe COVID-19 and sepsis, we provide some suggestions on how to manage three major complications - hyperinflammatory response, immunosuppression and hypovolemia in COVID-19 patients based on lessons from sepsis.

### Hyperinflammatory Response

The elevated levels of pro-inflammatory cytokines observed in severe COVID-19 cases (12, 44), suggest that keeping the immune system from making such an exacerbated response may improve clinical outcome. Hence, recommendations for management of severe inflammatory response syndrome (SIRS) in sepsis may benefit severe COVID-19 patients (45). In sepsis and septic shock, downregulating adverse inflammatory responses is a recommended adjunctive therapy, for which corticosteroids are used (45). Interestingly, six cohort studies confirmed that steroid use was associated with a better clinical outcome in patients with COVID-19 [reviewed in (46)]. However, two cohort studies reported negative outcomes - a higher risk of bloodstream infections (47) and a lower ten-day remission rate (48). Importantly, all the cohort studies had confounders, which limit their generalizability (46). Therefore, the use of immunosuppressive agents such as corticosteroids warrants extensive trials to confirm their actual benefits before recommending them for COVID-19 management. Perhaps, administration of immunosuppressive agents should be considered on a case-by-case basis and their use should be limited to the hyperinflammatory phase of COVID-19. Even the administration of immunosuppressive agents in the hyperinflammatory phase must be done with extreme caution to avoid predisposing patients to opportunistic infections (49).

As with sepsis, severe COVID-19 patients should be evaluated for SIRS. However, there are no specific laboratory tests for hyperinflammatory response, even for sepsis, that could be directly transposed to COVID-19. It is suggested that assessing a spectrum of parameters, such as erythrocyte sedimentation rate, C-reactive protein, plasma viscosity, IL-6, IL-8, procalcitonin, plasma ferritin, soluble urokinase plasminogen activator receptor and neutrophil gelatinase-associated lipocalin could provide insight on the patient’s hyperinflammatory status (50, 51). It is therefore necessary to develop superior molecular techniques to precisely assess the hyperinflammatory status. This could improve the management of COVID-19. Specifically, with such diagnostic techniques, it would be easier to know whether and when to administer an immunosuppressive agent to a patient.

### Immunosuppression

Accumulating evidence is implicating immunosuppression in severe COVID-19. Therefore, COVID-19 management should aim to reverse immunosuppression and prevent resultant opportunistic infections. Previous studies on the mechanism of immunosuppression during sepsis have implicated overexpression of programmed death-1 (PD-1), an immune checkpoint, and its ligand known as programmed death ligand-1 (PDL-1) (52). Overexpression of other immune checkpoints, such as cytotoxic T-lymphocyte antigen-4 and B- and T- lymphocyte attenuator, is also known to drive immune suppression (53). Furthermore, downregulation of HLA-DR, accelerated lymphocyte death or exhaustion, and clonal expansion of anti-inflammatory cells are all known correlates of immunesuppression during sepsis (53–55). Interestingly, blocking the immune checkpoints, particularly, PD-1/PDL-1, has been shown to restore immune competence, howbeit, preclinically (52, 53). Hence, these important immune-checkpoints remain attractive therapeutic targets that should be investigated for COVID-19 management. Other immune-boosting approaches should also be considered for managing immunosuppressed COVID-19 patients. This search should include vitamins, antioxidants, and other food supplements that are likely to boost immune response. It is plausible to target the immune-boosting therapies to severe phase when the immune system is compromised.

### Hypovolemia

Hypovolemia leading to hypotension, reduced venous return, low tissue perfusion and multiple organ dysfunction are associated with septic shock (56, 57). Hence, fluid resuscitation, with the aim of restoring intravascular volume, adequate tissue perfusion and organ homeostasis is often undertaken in septic shock management, howbeit, with caution against pulmonary fluid overload (56, 58). Fluid resuscitation could also be considered for the management of COVID-19, but following relevant guidelines regarding the type, volume and duration of the fluid (45). In septic shock patients presenting with persistent hypotension despite rational fluid resuscitation, vasopressors and inodilators have been recommended to restore tissue perfusion, systemic vascular resistance, cardiac output, and blood pressure (45).

Unlike sepsis, there is paucity of evidence of shock among COVID-19 patients (43). More studies comparing response of sepsis and COVID-19 patients are warranted to establish a suitable strategy for resuscitating hypovolemic COVID-19 patients. The proposed approaches are summarized in **Table 2**.

**Table 2:** Summary of approaches for managing sepsis conditions that could be explored for COVID-19 management.

Condition	Proposed approaches	Remark on whether approved by sepsis campaign alliance paper
Immunosuppression	Blocking of immune checkpoints, particularly, PD-1/PDL-1, to restore immune competence in COVID-19 patients	#
Hyperinflammatory response	Assessing parameters like, erythrocyte sedimentation rate, C-reactive protein, plasma viscosity, IL-6, IL-8, procalcitonin, plasma ferritin, soluble urokinase plasminogen activator receptor and neutrophil gelatinase-associated lipocalin could provide insight on the patient’s hyperinflammatory status	#
	Use of immune-boosting approaches such as vitamins, antioxidants, and other food supplements that are likely to boost immune response should also be considered for managing immunosuppressed COVID-19 patients	#
Hypovolemia leading to hypotension, low tissue perfusion and multiple organ dysfunction	Fluid resuscitation, with the aim of restoring intravascular volume, adequate tissue perfusion, and organ homeostasis	Weakly recommended
	Vasopressors and inodilators	Weakly recommended

^#^Surviving Sepsis Campaign document has not made express recommendations about the approaches proposed. Detailed research still required.

## Limitations

Although sepsis and COVID-19 have many striking parallels, a direct transposition of sepsis management to COVID-19 management should be taken with some pinch of caution. COVID-19 pathophysiology is not fully understood at the moment and appears to differ from person to person or from region to region. It is also important to note that sepsis remains a major global health burden, suggesting that not all its protocols are superior and must be evaluated further in the context of COVID-19. In fact, with the rapid and concerted efforts directed toward COVID-19, perhaps sepsis management may end up benefiting more from COVID-19.

## Concluding Remarks

The gravity of the COVID-19 pandemic requires a variety of solutions. As vaccine development continues, adapting existing treatment regimens from diseases with similar pathogenic pathways could help improve treatment outcomes and lower the mortality associated with SARS-CoV-2 infection. Here, we have discussed the parallels between COVID-19 and sepsis, focusing on their similarities with regards to immunopathogenesis and pathophysiology and offer suggestions on how sepsis condition might inform on management of three major complications in COVID-19. Further studies leveraging sepsis pathophysiology are warranted to help deal with COVID-19. On the flipside, considering the enormous efforts and resources directed toward COVID-19, sepsis management, which is still a major global health burden, may also benefit from COVID-19.

## Author Contributions

COO, NNN, KT, ALDZ, and OO wrote sections of the manuscript. YB and SD critically reviewed the draft. All authors contributed to the article and approved the submitted version.

## Funding

COO, KT, ALDZ, and OO were supported by WACCBIP-ACE PhD fellowship (ACE02-WACCBIP: Awandare) and a DELTAS Africa grant (DEL-15-007: Awandare). The DELTAS Africa Initiative is an independent funding scheme of the African Academy of Sciences (AAS)’s Alliance for Accelerating Excellence in Science in Africa (AESA) and supported by the New Partnership for Africa’s Development Planning and Coordinating Agency (NEPAD Agency) with funding from the Wellcome Trust (107755/Z/15/Z to Awandare) and the UK government. The views expressed in this publication are those of the author(s) and not necessarily those of AAS, NEPAD Agency, Wellcome Trust or the UK government.

## Conflict of Interest

The authors declare that the research was conducted in the absence of any commercial or financial relationships that could be construed as a potential conflict of interest.
